# Characteristics of clinical-pharmacological recommendations in psychiatry in Germany

**DOI:** 10.1177/00912174231177230

**Published:** 2023-05-16

**Authors:** Sebastian Schröder, Martin Schulze Westhoff, Tabea Pfister, Stefan Bleich, Felix Wedegärtner, Tillmann HC Krüger, Johannes Heck, Adrian Groh

**Affiliations:** 1 Department of Psychiatry, Social Psychiatry and Psychotherapy, 9177 Hannover Medical School, Hannover, Germany; 2 Institute for Clinical Pharmacology, 9177 Hannover Medical School, Hannover, Germany

**Keywords:** Clinical pharmacology, psychiatry, drug safety, interdisciplinarity, geriatric psychiatry

## Abstract

**Objective:**

Psychiatric patients in general, and elderly psychiatric patients in particular, are at risk of adverse drug reactions due to comorbidities and inappropriate polypharmacy. Interdisciplinary and clinical-pharmacologist-led medication reviews may contribute to medication safety in the field of psychiatry. In this study, we reported the frequency and characteristics of clinical-pharmacological recommendations in psychiatry, with a particular focus on geriatric psychiatry.

**Method:**

A clinical pharmacologist, in collaboration with the attending psychiatrists and a consulting neurologist, conducted interdisciplinary medication reviews in a general psychiatric ward with a geropsychiatric focus at a university hospital over a 25-week period. All clinical and pharmacological recommendations were recorded and evaluated.

**Results:**

A total of 316 recommendations were made during 374 medication reviews. Indications/contraindications of drugs were the most frequently discussed topics (59/316; 18.7 %), followed by dose reductions (37/316; 11.7 %), and temporary or permanent discontinuation of medications (36/316; 11.4 %). The most frequent recommendations for dose reduction **involved** benzodiazepines (9/37; 24.3 %). An unclear or absent indication was the most common reason for recommending temporary or permanent discontinuation of the medication (6/36; 16.7 %).

**Conclusion:**

Interdisciplinary clinical pharmacologist-led medication reviews represented a valuable contribution to medication management in psychiatric patients, particularly the elderly ones.

## Introduction

Psychiatric patients, especially elderly psychiatric patients, are susceptible to adverse drug reactions (ADRs) due to comorbidities and inappropriate polypharmacy.^1,2^ Inappropriate medication management can increase morbidity, mortality, and health economic costs.^3^

Therefore, clinical-pharmacological expertise is becoming increasingly important in the treatment of psychiatric patients. In other medical disciplines, participation of clinical pharmacologists (or clinical pharmacists, depending on the healthcare setting**)** in interdisciplinary ward rounds has already been established, eg. in internal medicine or surgery.^4,5^

Patients with severe mental illnesses are more likely to have somatic comorbidities and display higher mortality rates, resulting in a reduction in life expectancy by up to 12 years.^6^ Elderly psychiatric patients **are** additionally affected by age-related risk factors**,** such as sarcopenia, frailty, aging of the immune system (immunosenescence), and impaired organ function, leading to an increased risk of ADRs.^1,7^ Medication errors, drug–drug or drug–disease interactions, prescription of potentially inappropriate medications, potentially inappropriate duplicate prescriptions, and generally increased susceptibility to ADRs represent challenges in the treatment of elderly psychiatric patients.^8,9^

The aim of the present study was to analyze the frequency and characteristics of clinical-pharmacological recommendations made during interdisciplinary medication reviews over the course of nine months. This study was conducted in a general psychiatric ward with a geropsychiatric focus at a university. Our study sought to contribute to the improvement of drug and patient safety in (geriatric) psychiatry. Clinical-pharmacological recommendations for daily practice will be provided based on the results of this study.

## Method

### Ethics approval

This study was conducted in accordance with the World Medical Association Declaration of Helsinki and its amendments. As required by the Professional Code of Conduct of the local Medical Association, the local Ethics Committee was contacted. Based on an evaluation of the anonymized data, there were no ethical concerns, and the Ethics Committee waived the formal ethics vote.

### Study setting

This study was conducted in a general psychiatric ward with a geropsychiatric focus of a university hospital. Thus, the majority of patients in this study were elderly. In total, 374 medication reviews were performed for 149 patients across 25 visits (at weekly intervals) between February 2022 and October 2022. All the patients were inpatients. There were no specific exclusion criteria. Data were analyzed retrospectively and anonymized.

### Interdisciplinary medication reviews

Patient medications were reviewed by an interdisciplinary team of physicians comprising attending psychiatrists, a neurologist, and a clinical pharmacologist. The medication reviews covered the following topics (non-exhaustive list): checks for drug–drug interactions, correction of imprecise prescriptions or medication errors, (re-)evaluation of indications/contraindications, management of ADRs, and management of potentially inappropriate duplicate prescriptions (defined according to Heck et al.)^10,11^; management of potential prescription omissions; time point, frequency, or duration of drug administration; posology; and therapeutic drug monitoring. Patients’ medical histories, vital signs, laboratory results, and results from technical examinations (eg. imaging, microbiology, and cerebrospinal fluid analysis) were considered during medication reviews. Of note, the clinical pharmacologist and the neurologist only made recommendations during the medication reviews. The clinical decisions and the responsibility for the decisions remained with the treating psychiatrists.

### Statistics

Microsoft® Excel® 2019 (Redmond, Washington, USA) and IBM® SPSS® Statistics 28 (Armonk, New York, USA) were used for statistical analysis. Descriptive statistical methods were used to summarize the data. Quantitative variables were tested for normal distribution using the Shapiro–Wilk test. Normally distributed variables are presented as means ± standard deviations, while non-normally distributed variables are additionally displayed as medians with interquartile ranges (IQRs). Ranges (ie. minimum-to-maximum values) were reported for both normally and non-normally distributed variables. Categorical variables were presented as frequencies and percentages.

## Results

### Study population and medication reviews

The study population comprised 149 patients, for whom 374 medication reviews were performed, documented, and evaluated. The number of medication reviews per patient ranged from 1 to 18. The large number of medication reviews for some patients was mainly due to prolonged treatment duration (the average treatment duration in our study population was 64.2 ± 60.8 days), reflective of chronic and/or refractory disease processes. In addition, psychosocial reasons contributed to extended hospital stays in some patients. On average, 15.0 ± 3.3 medication reviews were conducted per visit date (range: 7 to 22 medication reviews per visit date).

Of 149 patients, 84 were women (56.4 %) and 65 were men (43.6 %). The mean age of the patients for whom medication reviews were conducted (n = 374) was 66.4 ± 15.0 years (median age 69 years; IQR: 57.75 to 78 years; range: 25 to 92 years).

Table 1 provides an overview of the psychiatric diagnoses and somatic comorbidities in the study population. The most frequent psychiatric disorder in the study population was depression (36.9 %; 55/149), whereas the most prevalent somatic comorbidity was arterial hypertension (44.3 %; 66/149). Notably, more than one-third (33.6 %; 50/149) of the study population had dementia.

**Table 1.** Characteristics of the study population (n = 149; mean age 66.4 ± 15.0 years, range 25–92 years).

Variables	n	%
Sex		
Female	84	56.4
Male	65	43.6
Psychiatric diagnoses^a^		
Depression^b^	55	36.9
Bipolar affective disorder^c^	11	7.4
Schizophrenia or schizophreniform disorder^d^	35	23.5
Mental and behavioral disorder due to use of alcohol, tobacco, or sedatives or hypnotics^e^	39	26.2
Dementia^f^	50	33.6
Delirium^g^	17	11.4
Other psychiatric disorder(s)	37	24.8
Somatic diagnoses^a^		
Arterial hypertension	66	44.3
Coronary heart disease	13	8.7
Chronic heart failure	17	11.4
Atrial fibrillation	15	10.1
Status post stroke	9	6.0
Type-2 diabetes mellitus	14	9.4
Chronic obstructive pulmonary disease	4	2.7
Hypothyroidism	13	8.7
Urinary tract infection	10	6.7
Other somatic disorder(s)	117	78.5

^a^Patients with more than one diagnosis:

^b^ICD-10 F32, F33;

^c^ICD-10 F31;

^d^ICD-10 F06.2, F2X;

^e^ICD-10 F10, F13, and F17;

^f^ICD-10 F00, F01, F02, and F03;

^g^ICD-10 F05. ICD-10: International Statistical Classification of Diseases and Related Health Problems, 10^th^revision.

The mean number of drugs taken per patient was 8.9 ± 3.9 (median 9 drugs; IQR: 6 to 11 drugs; range: 1 to 23 drugs).

### Clinical-pharmacological recommendations

**Figure 1.**
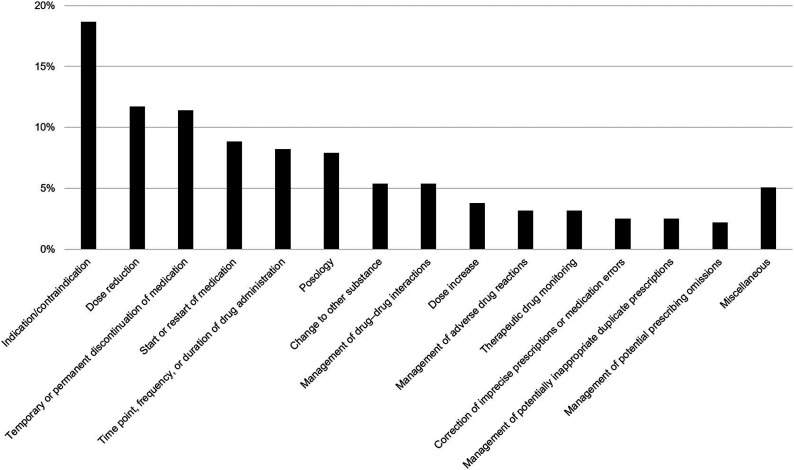
Categorization of clinical-pharmacological recommendations (n = 316). For numerical values and details on the different categories, please refer to Supplementary Table 1.

A total of 316 clinical-pharmacological recommendations were made during the 374 medication reviews (Figure 1 and Supplementary Table 1). Clinical-pharmacological recommendations most frequently centered on indications/contraindications of drugs (18.7 %; 59/316), followed by dose reductions (11.7 %; 37/316) and temporary or permanent discontinuation of drugs (11.4 %; 36/316).

Approximately one-quarter (24.3 %; 9/37) of dose reductions involved benzodiazepines. An unclear or absent indication was the most common reason for recommending temporary or permanent discontinuation of drugs (16.7 %; 6/36). Nine of 26 recommendations (34.6 %) in the category “time point, frequency, or duration of drug administration” involved once-daily administration (in lieu of multiple times per day) of drugs with long half-lives (eg. inhibitors of the renin–angiotensin–aldosterone system, torsemide, olanzapine, or escitalopram). Nearly one-quarter (24.0 %; 6/25) of dosing-related recommendations (other than dose increases or reductions) concerned appropriate dosing of parenteral (eg. tinzaparin) and oral (eg. apixaban) anticoagulants. Switching from metoprolol tartrate to metoprolol succinate due to more favorable pharmacokinetics of the latter, especially in long-term treatment, was the most frequent advice in the category “change to other substance” (17.6 %; 3/17). The largest proportion of recommendations pertaining to the management of drug–drug interactions concerned the risk of serotonin syndrome (29.4 %; 5/17). Half (4/8) of the corrections for inaccurate prescriptions or medication errors involved units of levothyroxine (confusion of micrograms (µg) with milligrams (mg)). Half (4/8) of the recommendations dealing with potentially inappropriate duplicate prescriptions involved combinations of sedative agents (eg. benzodiazepines, Z-drugs, or low-potency first-generation antipsychotics).

## Discussion

Pharmacotherapy is the treatment of choice for several psychiatric disorders, particularly affective and psychotic disorders.^12^ Elderly people with mental illnesses are often additionally suffering from somatic comorbidities, which usually increase the number of drugs required for treatment. In our study the median age of the patients was 69 years and the median number of drugs was 9, reflective of the study population’s multimorbidity (Table 1).

Benzodiazepines accounted for nearly one-quarter of the recommended dose reduction. This is consistent with the clinical consensus that benzodiazepine doses should generally be lower in elderly patients than in younger individuals (approximately one-half) and that benzodiazepines should be discontinued gradually rather than abruptly because of the risk of withdrawal symptoms (including delirium).^13,14^ Benzodiazepines are often prescribed for anxiety disorders, which are common in the elderly and usually respond well to benzodiazepines in the short term.^15^ If possible, benzodiazepines should be avoided in patients aged > 65 years;^16^ however, this is often not feasible in clinical practice. Notwithstanding, it is of utmost importance to keep benzodiazepine doses as low as possible in elderly psychiatric patients, as this population is particularly susceptible to the development of ADRs, such as sedation, cognitive impairment, falls, or even paradoxical reactions (eg. agitation).^13,14^

Unclear or absent indications were the leading cause of temporary or permanent discontinuation of drugs. The cessation of clinically unnecessary drugs may reduce the risk of ADRs, promote therapy adherence, and reduce healthcare expenditures.^17^

Tinzaparin and risperidone most frequently accounted for medication (re-)starts. On the one hand, anticoagulants are frequently associated with ADRs (especially bleedings) in elderly patients. On the other hand, due to the increased risk of thromboembolic events, thromboprophylaxis is recommended for immobile patients and is widely used during inpatient stay. Treatment with anticoagulants may protect patients from thromboembolic events, but at the same time exposes them to the risk of bleeding, with potentially serious outcomes.^18^ This demonstrates that diligent benefit–risk analyses are indispensable prior to the initiation of anticoagulant treatment. In such therapeutic dilemmas, consultation with a clinical pharmacologist may be advantageous for optimizing medication safety. A systematic review with meta-analysis showed that the risk of hemorrhagic events and thrombosis can be significantly reduced by pharmacist-based interventions with special emphasis on anticoagulation.^19^ For example, a pharmacist-led anticoagulation service has been introduced in several hospitals, with pharmacists helping to determine the dose of anticoagulation, monitor laboratory values, and educate patients.^20^

Risperidone has been approved for the treatment of agitation and aggression in Alzheimer’s dementia, a common neurodegenerative disease in elderly psychiatric patients, but only for six weeks. Therefore, risperidone prescriptions should be reviewed critically.^21^ In general, the inappropriate use of antipsychotics in elderly patients can entail various ADRs, such as sedation, cognitive impairment, delirium, and fall-related fractures.^22^ Moreover, the United States Food and Drug Administration issued a boxed warning in 2008, stating that elderly patients with dementia-related psychosis and treated with antipsychotics are at an increased risk of death.^23^ However, this boxed warning must not be understood as a contraindication, and antipsychotics can still be prescribed to elderly psychiatric patients, also outside their approved indications, if considered clinically necessary by the treating physicians.^24^

More than one-third of clinical-pharmacological recommendations in the category “time point, frequency, or duration of drug administration” concerned the once-daily administration (in lieu of multiple applications per day) of drugs with long half-lives. In addition, the use of combination preparations can also reduce patients’ “pill count.” The number of drug applications per day and the “pill count” should generally be as low as possible to promote therapy adherence.^25^

Nearly one-quarter of dosing-related recommendations refer to anticoagulants. Observing dosage recommendations for direct oral anticoagulants is extremely important because a recent meta-analysis demonstrated that off-label underdosing of direct oral anticoagulants is associated with increased all-cause mortality in patients with atrial fibrillation, without (positive) effect on bleeding outcomes.^26^

A considerable proportion of drug–drug interactions were related to the risk of serotonin syndrome. This ADR is rare but potentially life-threatening and should, therefore, always be considered by psychiatrists when prescribing medications with a serotonergic mode of action.^27^

Recommendations for dose increase were most frequently made for pregabalin and atorvastatin. This reflects the pharmacoepidemiologic trend that pregabalin is not only used for its anticonvulsant properties but also for the treatment of neuropathic pain and anxiety disorders.^28^ However, the potential of pregabalin for sedation and dependency development should be observed. β-Hydroxy β-methylglutaryl coenzyme A reductase inhibitors (“statins”) are the most commonly prescribed lipid-lowering medications and, intriguingly, also seem to possibly exert a positive effect on depressive disorders,^29^ which represented the most common psychiatric disorder in our study population.

Therapeutic drug monitoring (TDM) is particularly useful for drugs within narrow therapeutic ranges. This explains why 40% and 20% of the therapeutic drug monitoring recommendations referred to clozapine and lithium, respectively. In addition to its undisputed relevance in lithium therapy, TDM is extremely important to achieve adequate clozapine serum levels, as a minimum serum level of 350 ng/ml is required to achieve the desired therapeutic effect of clozapine.^30^

In the present study, we applied the novel categorization of duplicate prescriptions by Heck et al., which fundamentally distinguishes between appropriate and potentially inappropriate duplicate prescriptions.^10,11^ In this study, potentially inappropriate duplicate prescriptions mostly involved combinations of sedatives such as benzodiazepines, Z-drugs, and low-potency first-generation antipsychotics. This corroborates previous findings by colleagues, who also found sedatives to be major contributors to potentially inappropriate duplicate prescriptions in patients with psychiatric disorders.^31^

Half of the corrections for imprecise prescriptions or medication errors involved the use of levothyroxine. Incorrect use of the unit “mg” instead of “µg” formally results in a 1000-fold overdose of levothyroxine. Fortunately, in clinical practice, the risk of medication errors associated with thyroid hormones appears manageable, as no preparations in the milligram range exist. Nevertheless, for preventive patient protection, particular care should always be exercised when prescribing medications.

Potential prescribing omissions in our study were mainly related to drugs for the treatment of cardiovascular diseases (eg. antiplatelet agents and β-hydroxy β-methylglutaryl coenzyme A reductase inhibitors), a finding which is consistent with a report by Parodi Lopez and co-workers.^32^

To date, most interdisciplinary medication reviews have been conducted by clinical pharmacists, not by clinical pharmacologists. For example, in a study by Molist-Brunet et al., polypharmacy and prescription rates of sedatives were significantly reduced by conducting medication reviews on a geriatric inpatient population.^33^ Stuhec and colleagues found that medication reviews by clinical pharmacists can increase prescribing quality in geropsychiatric outpatients.^34^ Other studies have shown that medication reviews may reduce the incidence of ADRs.^35,36^ In contrast, our study assessed the participation of a clinical pharmacologist and neurologist in the interdisciplinary management of psychiatric patients. Data on medication reviews in a psychiatric context are generally sparse. Our analysis laid the groundwork for future prospective studies that may investigate the clinical benefit of clinical pharmacologist-led medication reviews.

## Study limitations

In summary, interdisciplinary clinical pharmacologist-led medication reviews provide an opportunity to detect and resolve medication-related problems. However, the involvement of a clinical pharmacologist (as well as the participation of a neurologist, as conducted in our study) is costly, time-consuming, and difficult to implement in non-university hospitals or countries with less affluent healthcare systems. Hence, our results are not universally applicable to other healthcare settings.

Moreover, it must be critically annotated that the overall number of clinical-pharmacological recommendations in our study was relatively low (316 recommendations in 374 medication reviews). However, the number of recommendations did not reflect their quality. Each ADR that can be prevented as a result of the engagement of a clinical pharmacologist and neurologist may reduce healthcare expenditures, although it was not possible to assess how many ADRs were prevented as a result of our interdisciplinary medication reviews because of the retrospective nature of our study. Future prospective studies should address this research question.

The limitations of our investigation arise from its monocentric setting and lack of a control group. Therefore, it remains unclear whether our interdisciplinary medication review improved patient care. Patients’ preexisting conditions were extracted retrospectively from medical records, which may be subject to bias. Furthermore, it was not incentivized to document every recommendation, which may have led to underreporting. Also, competing patient care demands may have limited the ability of physicians to document.

In the future, direct patient contact during our interdisciplinary medication reviews is envisaged. Regarding further scientific evaluation, a randomized clinical trial with an intervention group and a control group is being devised to identify whether the quality of treatment and pharmacotherapy safety in psychiatric patients can be improved by an interdisciplinary, clinical pharmacologist-led medication review.

## Conclusions

Interdisciplinary, clinical pharmacologist-led medication reviews can represent a valuable tool to optimize the management of psychiatric patients, particularly those who are elderly. During interdisciplinary medication reviews in psychiatry, particular attention should be paid to sedative agents (especially their indication, age-adapted dosing, and avoidance of potentially inappropriate duplicate prescriptions), therapeutic drug monitoring (especially for clozapine and lithium), and use of correct units (prescription of levothyroxine in micrograms, not milligrams). Metoprolol succinate should be preferred to metoprolol tartrate in long-term treatment due to more favorable pharmacokinetics. If clinically indicated, drugs for the treatment of cardiovascular diseases (especially antiplatelet agents and β-hydroxy β-methylglutaryl coenzyme A reductase inhibitors) should be prescribed to reduce potential prescribing omissions. Drugs with long half-lives (eg. inhibitors of the renin–angiotensin–aldosterone system, torsemide, olanzapine, or escitalopram) should preferably be prescribed once per day (instead of multiple times per day) to promote patients’ therapy adherence. Physicians should adhere to the dosing recommendations of direct oral anticoagulants as laid down in the summaries of product characteristics, since both over- and underdosing of direct oral anticoagulants can be detrimental to patients.

## Supplemental Material

Supplemental Material - Characteristics of clinical-pharmacological recommendations in psychiatry in Germany Supplemental Material for Characteristics of clinical-pharmacological recommendations in psychiatry in Germany by Sebastian Schröder, Martin Schulze Westhoff, Tabea Pfister, Stefan Bleich, Felix Wedegärtner, Tillmann HC Krüger, Johannes Heck, and Adrian Groh in The International Journal of Psychiatry in Medicine
