# Supplementary material for: Characteristics of clinical-pharmacological recommendations in psychiatry in Germany
Source: Int J Psychiatry Med. 2023 May 16;59(3):393–405. doi: 10.1177/00912174231177230 (PMC11044510; doi:10.1177/00912174231177230)
Supplement: Supplemental Material - Characteristics of clinical-pharmacological recommendations in psychiatry in Germany [file sj-pdf-1-ijp-10.1177_00912174231177230.pdf]

SUPPLEMENTARY TABLE 2 Categorization of clinical-pharmacological recommendations (n = 316)

**Category**	**n**	**%**
Indication[a]/contraindication	59	18.7
Dose reduction	37	11.7
Temporary or permanent discontinuation of medication	36	11.4
Start or restart of medication	28	8.9
Time point, frequency, or duration of drug administration	26	8.2
Posology[b]	25	7.9
Change to other substance[c]	17	5.4
Management of drug–drug interactions	17	5.4
Dose increase	12	3.8
Management of adverse drug reactions	10	3.2
Therapeutic drug monitoring	10	3.2
Correction of imprecise prescriptions or medication errors	8	2.5
Management of potentially inappropriate duplicate prescriptions[d]	8	2.5
Management of potential prescribing omissions	7	2.2
Miscellaneous	16	5.1

[a]not related to potential prescribing omissions
[b]other than dose increase or reduction
[c]not related to the management of adverse drug reactions
[d]according to Heck et al. [10][11]
